# The COVID-19 Pandemic and the Incidence of the Non-COVID-19 Pneumonia in Adults

**DOI:** 10.3389/fmed.2021.737999

**Published:** 2021-11-11

**Authors:** Chienhsiu Huang

**Affiliations:** Division of Chest Medicine, Department of Internal Medicine, Dalin Tzu Chi Hospital, Buddhist Tzu Chi Medical Foundation, Chiayi, Taiwan

**Keywords:** COVID-19, community acquired pneumonia, hospital acquired pneumonia, nursing home acquired pneumonia, pandemic

## Abstract

**Introduction:** The coronavirus disease 2019 (COVID-19) lockdown strategies were associated with a significant decrease in the common respiratory viral diseases and decreased the need for hospitalization among children in the COVID-19 outbreak. However, the trend of non-COVID-19 pneumonia in adult people remains uncertain. Our aim is to assess the impact of the COVID-19 pandemic on the incidence of the non-COVID-19 pneumonia in adult people and understand whether the substantial decrease in pneumonia cases is the same as the decline in the incidence of respiratory viral disease activity.

**Methods:** We conducted a retrospective analysis of adult patients presenting with pneumonia from January 2019 to December 2020. Details on all the demographics of the patient of pneumonia, hospital course details, prior admission history within 3 months, respiratory culture, and antibiotics sensitivity test were also obtained.

**Results:** The number of adult patients with community-acquired pneumonia in 2020 was lower than that in 2019, which decreased by 74 patients in 2020. The decreasing number of patients with community-acquired pneumonia between 2019 and 2020 was from −13.9% in January to March 2020 to −39.7% in October to December 2020. The decreasing number of patients with community-acquired pneumonia between 2019 and 2020 was from −14.8% in the youngest cohort to −28.7% in those aged ≥85 years. The number of reduced patients with community-acquired pneumonia is greater in late seasons and older age, respectively. The number of adult patients with hospital-acquired pneumonia in 2020 was lower than that in 2019, which decreased by 23 patients in 2020. The decreasing number of patients with hospital-acquired pneumonia between 2019 and 2020 was from −20.0% in January to March 2020 to −52.4% in October to December 2020. The decreasing number of patients with hospital-acquired pneumonia between 2019 and 2020 was from 0% in the youngest cohort to −45.6% in those aged ≥ 85 years. The number of reduced patients with hospital-acquired pneumonia is greater in late seasons and older age, respectively.

**Conclusion:** Interventions applied to control the COVID-19 pandemic were effective not only in substantial changes in the seasonal influenza activity, but also in decreasing adult pneumonia cases.

## Introduction

**Figure 1:**
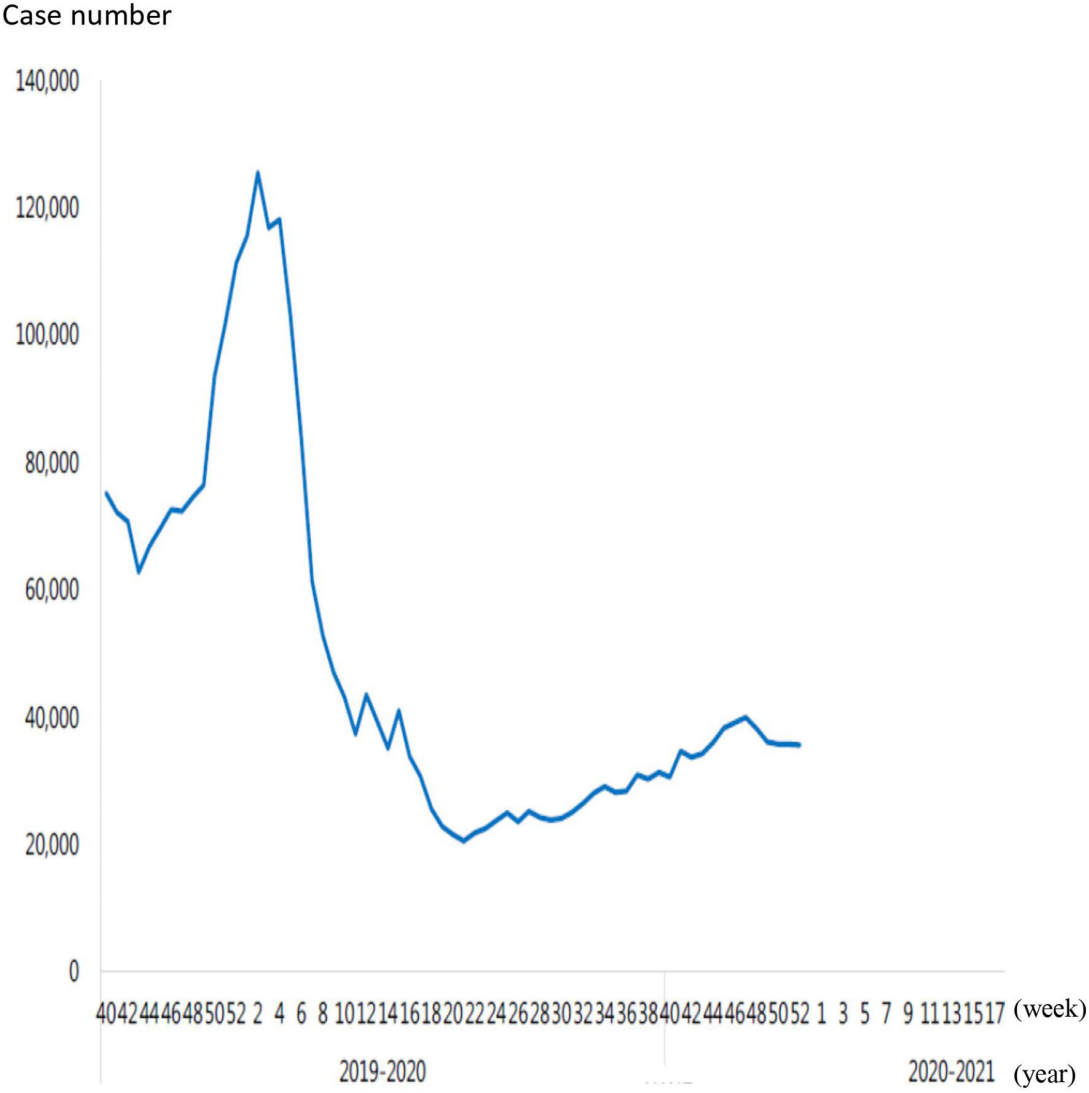
The total number of outpatient and emergency visits for influenza-like illness in 2019, 2020 and 2021.

The coronavirus disease 2019 (COVID-19) outbreak first occurred in December 2019 in Wuhan, Hubei Province, China. Within 3 months, the COVID-19 had quickly spread worldwide. The incidence of COVID-19 then substantially increased and it was declared a pandemic by the WHO on March 11, 2020 (1). The governments of various countries worldwide have widely promoted several measures to prevent the COVID-19 outbreak such as education on hand hygiene and cough etiquette, staying at home if respiratory symptoms persist, using a mask in public places, social distancing, travel restrictions, and closure of schools. The government of Taiwan undertook several rapid measures since January 2020 to prevent the COVID-19 outbreak such as border control, case identification, quarantine of suspicious cases, and travel restriction. As of March 14, 2020, people returning directly to Taiwan from other countries had to stay under quarantine at home for 14 days. Taiwanese citizens were advised to avoid all the non-essential travels. All the healthcare workers were prohibited from going abroad. The Taiwan Centers for Disease Control announced social distancing measures to encourage the general public to maintain social etiquette and to cancel or postpone conferences and social gatherings. The rules specified separate social distancing standards for restaurants, school campuses, offices, mass transport, supermarkets, and special institutions such as long-term care facilities and prisons. The use of face masks during all the instances of close contact was widely recommended throughout the country (2). The following strategies were implemented to prevent the COVID-19 outbreak in our hospital: (1) Only three entrance doors to the hospital buildings were opened including the entrance door of the emergency room (ER), (2) Only one car entrance was allowed during daytime, (3) Body temperature of individuals entering the premises was checked by using an IR thermal camera, (4) We checked the National Health Insurance identification card data of all the visitors who entered our hospital when the confirmed COVID-19 cases were increasing in the community, (5) All the people had to wear surgical masks and were sprayed with 75% alcohol for hand hygiene when they entered the hospital, (6) All the febrile hospital visitors were transferred to the ER for further evaluation and management, (7) All the persons with respiratory symptoms had a travel history of epidemic countries or closely contact with patients with the confirmed COVID-19 diagnosis and were transferred to the ER for further evaluation and management, and (8) The number and time of inpatient visitor caregivers and visitors were restricted. Moreover, the implementation period of government-sponsored influenza vaccination was from October 2019 in Taiwan. The total number of outpatient and ER visits for influenza-like illness decreased since February 2020, which continued to decrease each week. Seasonal influenza activity continued to reduce to its lowest point in April 2020 (Figure 1) (https://www.cdc.gov.tw/En/File/Get/a-VtY5lEvxa8VSLx9KN-7A). The COVID-19 lockdown strategies were associated with a significant decrease in infectious diseases disseminated through the airborne or fecal–oral transmissions (3). In Korea, efforts to stimulate the national response not only decreased the spread of the COVID-19, but also substantially decreased seasonal influenza activity (4). The incidence of hospitalization of young children owing to respiratory syncytial virus and acute respiratory infections dramatically decreased after implementing social distancing in the Alaskan population (5). Social distancing and other lockdown strategies effectively impeded the spread of common respiratory viral diseases and decreased the need for hospitalization among children in Finland (6). Winter surveillance data from Australia and Japan also demonstrated a decrease in seasonal influenza activity compared with previous seasons (7, 8). Thus, a decline in the incidence of respiratory viral diseases was reported following the COVID-19 outbreak. However, the trend of the non-COVID-19 pneumonia in adults remains uncertain. Therefore, this study aimed to assess the impact of the COVID-19 pandemic on the incidence of pneumonia in adult people and understand whether the substantial decrease in pneumonia cases correlated with the decline in the incidence of respiratory viral disease activity.

## Methods

We conducted a retrospective analysis of all the patients (age > 18 years) presenting with pulmonary infection who were admitted to the Dalin Tzu Chi Hospital between January 2019 and December 2020. Patients were diagnosed with pulmonary infection if their clinical symptoms and signs indicated infection and their chest radiology displayed a new acute pulmonary infiltration. Community-acquired pneumonia (CAP) was defined as a pulmonary parenchymal infection in the patients who acquire the condition in the community. Hospital-acquired pneumonia (HAP) was defined as a pulmonary parenchymal infection in the patients who acquire the condition at least 48 h after the hospital admission or within 14 days of hospital discharge (9). All the patients with HAP in this study were diagnosed with pulmonary parenchymal infection within 14 days of hospital discharge.

Data were obtained from the clinical information system of hospital, microbiology laboratory report system, and medical chart review. Details on all the CAP and HAP (excluding pulmonary tuberculosis and pulmonary fungal infection), demographics of the patient, hospital course details, prior admission history within 3 months, respiratory culture, and antibiotics sensitivity test were also obtained.

Respiratory secretions were collected for culture. Bacterial cultures of respiratory secretions and examination for antibiotic susceptibility were performed by using the VITEK 2 system with the ASTGN87 cards (bioMérieux, Marcy-l'Étoile, France, UK) based on the Clinical and Laboratory Standards Institute interpretive criteria M100-S25. Respiratory secretions were cultured on chocolate agar, blood agar, eosin methylene blue agar, and colistin–nalidixic acid agar plates immediately after collection. Gram staining and microscopy were performed. Identification cards were inoculated with microorganism suspensions by using an integrated vacuum apparatus. A test tube containing the microorganism suspension was placed into a cassette. One identification card for each Gram-negative card, Gram-positive card, and VITEK 2 AST-N322 (for susceptibility testing against specified antimicrobial agents) was placed in the neighboring slot along with the transfer tube and corresponding suspension tube. Calculations were performed by using raw data and compared with thresholds to determine reactions for each test.

### Statistical Analysis

Continuous variables were expressed as mean ± SD or median (range), whereas categorical variables were expressed as frequencies and percentages. The trend in change of pneumonia cases was analyzed by linear-by-linear association by using the chi-squared test. All the statistical analyses were conducted by using the Statistical Package for the Social Sciences (SPSS) for Windows (version 17.0, SPSS Incorporation, Chicago, Illinois, USA). *p* < 0.05 was considered as statistically significant.

## Results

A total of 704 patients with pulmonary infection were admitted to the Dalin Tzu Chi Hospital during the study period including 574 patients with CAP, 121 patients with HAP, 7 patients with pulmonary tuberculosis, and 2 patients with pulmonary fungal infection.

### Community-Acquired Pneumonia

The number of adult patients with CAP who were admitted to our hospital between January and December 2020 was compared with those from the same period in 2019. A total of 324 patients were diagnosed with CAP in 2019 (including 67 nursing home residents) and a total of 250 patients were diagnosed with CAP in 2020 (including 67 nursing home residents). No significant difference was observed in the number of nursing home residents with CAP between 2019 and 2020 (*p* = 0.091). We further divided these patients with CAP into four groups based on seasons (January to March, April to June, July to September, and October to December) as shown in Table 1. It showed that the number of patients with CAP in 2020 was lower than that the number of patients with CAP in 2019, which decreased by 74 patients (−22.9%) in 2020. The decreasing number of patients with CAP between 2019 and 2020 increased with season from −13.9% in January to March 2020 to −39.7% in October to December 2020 (*p* = 0.001; *M*^2^ = 11.899). This study explored the influence of increasing age on the number of patients with CAP by comparing the following five age cohorts: <55, 55–64, 65–74, 75–84, and ≥85 years in 2 years as shown in Table 1. The decreasing number of patients with CAP between 2019 and 2020 increased with age cohorts from −14.8% in the youngest cohort to −28.7% in those aged ≥85 years (*p* = 0.042; *M*^2^ = 4.256). In 2019, 136 patients with CAP were detected with pneumonia pathogens with the *Streptococci* group (19.1%) being the most common followed by *Pseudomonas aeruginosa* (*P. aeruginosa*) (17.6%) and *Klebsiella pneumoniae* (*K. pneumoniae*) (11.0%), whereas in 2020, 114 patients with CAP were detected with pneumonia pathogens with *K. pneumoniae* (21.1%) being the most common followed by *P. aeruginosa* (14.9%) and *Escherichia coli* (14.0%) (Table 2).

**Table 1:** Community-acquired pneumonia patients requiring hospitalization, by season and age cohorts.

	**Patients No (2019)**	**Patients No (2020)**	**Patients No (2019–2020)/2019**
**Season**			
January to March	79 (24.4%)	68 (27.2%)	11 (13.9%)
April to June	79 (24.4%)	63 (25.2%)	16 (20.3%)
July to September	93 (28.7%)	75 (30.0%)	18 (19.4%)
October to December	73 (22.5%)	44 (17.6%)	29 (39.7%)
Age cohorts^*^			
Age <55 Y/O, No (%)	27 (8.3%)	23 (9.2%)	4 (14.8%)
Age 55–64 Y/O, No (%)	38 (11.7%)	33 (13.2%)	5 (13.2%)
Age 65–74 Y/O, No (%)	51 (15.8%)	39 (15.6%)	12 (23.5%)
Age 75–84 Y/O, No (%)	100 (30.9%)	78 (31.2%)	22 (22.0%)
Age ≥ 85 Y/O, No (%)	108 (33.3%)	77 (30.85)	31 (28.7%)
Total	324	250	74 (22.9%)

* *The decreasing number of CAP patients between 2019 and 2020 increased with age cohorts, from −14.8% in the youngest cohort to −28.7% in those aged ≥85 years (P = 0.042; M^2^ = 4.256)*.

*No, number; Y/O, years old*.

**Table 2:** Identified pathogens in community-acquired pneumonia patients.

	**Patients No (2019)**	**Patients No (2020)**
Streptococci group (%)	26 (19.1%)	9 (7.9%)^*^
*Pseudomonas aeruginosa* (%)	24 (17.6%)	17 (14.9%)^#^
*Klebsiella pneumoniae* (%)	15 (11.0%)	24 (21.1%)
*Staphylococcus aureus* (MRSA) (%)	14 (10.3%)	5 (4.4%)
*Escherichia coli* (%)	11 (8.1%)	16 (14.0%)
*Haemophilus influenzae* (%)	10 (7.4%)	6 (5.3%)
*Acinetobacter baumannii* (CRAB) (%)	8	6
*Acinetobacter baumannii* (%)	6	3
*Klebsiella pneumoniae* (CRKP) (%)	3	4
*Stenotrophomonas maltophilia* (%)	2	1
*Pseudomonas aeruginosa* (CRPA) (%)	1	6
*Staphylococcus aureus* (MSSA) (%)	3	5
Influenza (%)	3	0
*Proteus mirabilis* (%)	2	7 (6.1%)
Others (%)	8	5
Total	136	114

* *There is significant different between 2019 and 2020. (P = 0.016, OR = 0.363, 95% confident interval: 0.162–0.810)*.

# *There is significant different between 2019 and 2020. (P = 0.036, OR = 2.151, 95% confident interval: 1.068–4.334)*.

*No, number; MSSA, methicillin sensitive staphylococcus aureus; MRSA, methicillin resistant staphylococcus aureus; CRAB, carbapenem resistant Acinetobacter baumannii; CRKP, carbapenem resistant Klebsiella pneumoniae; CRPA, carbapenem resistant Pseudomonas aeruginosa*.

### Hospital-Acquired Pneumonia

The number of adult patients with HAP who were admitted to our hospital from January to December 2020 was compared with those from the same period in 2019. A total of 72 patients were diagnosed with HAP in 2019 (including 23 nursing home residents) and a total of 49 patients were diagnosed with HAP in 2020 (including 19 nursing home residents). No significant difference in the number of nursing home residents with HAP was observed between 2019 and 2020 (*p* = 0.445). These patients were further divided into four groups based on seasons (January to March, April to June, July to September, and October to December) as shown in Table 3. It showed that the number of patients with HAP in 2020 was lower than that the number of patients with HAP in 2019, which decreased by 23 patients (−31.9%) in 2020. The decreasing number of patients with HAP between 2019 and 2020 increased with season from −20.0% in January to March 2020 to −52.4% in October to December 2020 (*p* = 0.016; *M*^2^ = 5.865). This study explored the influence of increasing age on the number of patients with HAP by comparing the following five age cohorts: <55, 55–64, 65–74, 75–84, and ≥85 years during the study period as shown in Table 3. The decreasing number of patients with HAP between 2019 and 2020 increased with age from 0% in the youngest cohort to −45.6% in the oldest cohort (*p* = 0.002; *M*^2^ = 8.955). In 2019, 40 patients with HAP were detected with pneumonia pathogens with *P. aeruginosa* (32.5%) being the most common followed by *Streptococci* group (15.0%) and *Acinetobacter baumannii* (*A. baumannii*) (15.0%), whereas in 2020, 21 patients with HAP were detected with pneumonia pathogens with *P. aeruginosa* (14.3%), *A. baumannii* (14.3%), carbapenem-resistant *A. baumannii* (CRAB) (14.3%), and methicillin-resistant *Staphylococcus aureus* (MRSA) (14.3%) (Table 4).

**Table 3:** Hospital-acquired pneumonia patients requiring hospitalization, by season and age cohorts.

	**Patients No (2019)**	**Patients No (2020)**	**Patients No (2019–2020)/2019**
**Season**			
January to March	15 (20.8%)	12 (24.5%)	3 (20.0%)
April to June	15 (20.8%)	13 (26.5%)	2 (13.3%)
July to September	21 (29.2%)	14 (28.6%)	7 (33.3%)
October to December	21 (29.2%)	10(20.4%)	11 (52.4%)
Age cohorts^*^			
Age <55 Y/O, No (%)	1 (1.40%)	1 (2.0%)	0 (0.00%)
Age 55–64 Y/O, No (%)	10 (13.9%)	10 (20.4%)	0 (0.00%)
Age 65–74 Y/O, No (%)	10 (13.9%)	9 (18.4%)	1 (10.0%)
Age 75–84 Y/O, No (%)	29 (40.3%)	17 (34.7%)	12 (41.4%)
Age ≥ 85 Y/O, No (%)	22 (30.5%)	12 (24.5)	10 (45.6%)
Total	72	49	23 (31.9%)

* *The decreasing number of patients with HAP between 2019 and 2020 increased with age cohort, from 0% in the youngest cohort to −45.6% in those aged ≥85 years (P = 0.002; M^2^ = 8.955)*.

*No, number; Y/O, years old*.

**Table 4:** Identified pathogens in hospital- acquired pneumonia patients.

	**Patients No (2019)**	**Patients No (2020)**
*Pseudomonas aeruginosa* (%)	13 (32.5%)	3 (14.3%)
*Streptococci* group (%)	6 (15.0%)	1 (4.8%)
*Acinetobacter baumannii* (%)	6 (15.0%)	3 (14.3%)
*Staphylococcus aureus* (MRSA) (%)	4 (10.0%)	3 (14.3%)
*Stenotrophomonas maltophilia* (%)	2 (5.0%)	2 (9.5%)
*Pseudomonas aeruginosa* (CRPA) (%)	2 (5.0%)	0
*Acinetobacter baumannii* (CRAB) (%)	1	3 (14.3%)
*Klebsiella pneumoniae* (%)	1	1 (4.8%)
*Klebsiella pneumoniae* (CRKP) (%)	1	0
Others (%)	4	5
Total	40	21

*No, number; MRSA, methicillin resistant staphylococcus aureus; CRAB, carbapenem resistant Acinetobacter baumannii; CRKP, carbapenem resistant Klebsiella pneumoniae; CRPA, carbapenem resistant Pseudomonas aeruginosa*.

## Discussion

The reduction in the incidence of viral diseases may be attributed to the COVID-19 preventive measures. However, no study has investigated the impact of preventive infection control measures in reducing the incidence of bacterial pneumonia. To the best of our knowledge, viral infection can trigger the development of bacterial pneumonia. Weinberger et al. reported a significant increase in pneumonia-related hospitalizations during the 2009–2010 H1N1 influenza pandemic.

Conversely, this study showed that the number of patients with CAP reduced by an average of 22.9% and that the number of patients with HAP reduced by an average of 31.9% in 1 year. The longer the number of days, the more the number of patients will decrease. To compare October to December season, the number of patients with CAP reduced by 39.7% in 2020 and the number of patients with HAP reduced by 52.4% in 2020. With respect to the age of the patients, the older the age, the greater the reduction in the number of patients in both the CAP and HAP groups. The strategy to prevent the COVID-19 outbreak has also been extremely effective in promoting the protection of the public from pneumonia, especially the elderly people. With regard to nursing home residents, the number of pneumonia did not significantly decrease in both the CAP and HAP groups during the COVID-19 outbreak period. Several modifiable factors were related to nursing home resident pneumonia such as sedative medications, tube feeding, depression, reduced level of consciousness, several chronic diseases, malnutrition, mobility limitation, poor oral hygiene, and dysphagia (10–17). Therefore, strategies to prevent the COVID-19 outbreak did not provide protection to nursing home residents against pneumonia.

Identification of the pneumonia pathogen is challenging. In the most pneumonia studies, between 35 and 70% of participants had no pathogen identified (18). The literatures of Taiwan showed that the percentage of unknown infectious pathogen of three pneumonia series was 25, 28, and 50%, respectively (19–21). The unknown infectious pathogen was 44.4% in 2019 and 45.2% in 2020 in our series, which was similar as literature reports.

With respect to CAP pathogens, the incidence of patients infected with the *Streptococci* group in this study showed a decreasing trend in 2020, which could be related to the decreased influenza activity in Taiwan. McCullers et al. showed that influenza infections increase the risk of developing secondary bacterial disease, particularly with streptococcal pneumonia (22). Daniel et al. reported a significant increase in hospitalizations owing to pneumococcal infections that corresponded to the timing of highest pandemic influenza activity (23). Besides, the number of *K. pneumonia* infections in this study increased in 2020. Lauderdale et al. showed that *S. pneumoniae* is the most common pathogen of adult patients with CAP requiring hospitalization and gram-negative bacilli, particularly *K. pneumoniae*, also play an important role in the infections of hospitalized patients with CAP in Taiwan (24). A prospective observational study of adult patients with CAP in 14 hospitals of eight Asian countries (including Taiwan) in 2008 displayed that *S. pneumoniae* was the most common isolate followed by *K. pneumoniae* and *Haemophilus influenzae* (25). A significant decrease in hospitalizations owing to pneumococcal infections in this study corresponded to the lowest influenza activity during the COVID-19 pandemic; however, a significant increase in hospitalizations owing to *K. pneumoniae* infections was observed in Taiwan. No difference was observed in the pathogens that caused HAP between 2019 and 2020, which may be owing to the extremely small number of HAP in this study.

More than 29 viruses have been linked to pneumonia. Among viral pneumonia, influenza virus remains the most common causes of viral pneumonia in adult. The ways for respiratory virus detection included isolation of the virus by viral culture, antigen detection in respiratory secretions, and reverse transcriptase-PCR in respiratory secretions (26). Influenza PCR was performed for intensive care unit patient who was suspected influenza pneumonia in our hospital. Therefore, there were only three influenza virus pneumonia cases in our series. According to the study by Peto et al., the viral pneumonia accounted for 9.8% of hospitalized CAP in Asian studies and 10.9% of hospitalized CAP in European studies. There were many studies on CAP in the literature and none of these series included viral pneumonia (27–30).

Only one rapid communication discussed the issue of the COVID-19 pandemic and the incidence of the non-COVID-19 pneumonia in the literature. Yamamoto et al. reported that the number of patients with CAP began to decrease in February 2020. The number was significantly lower than those during the same period in the last 3 years. The author thought that these COVID-19 outbreak measures might have indirectly contributed to a decrease in the number of cases through the prevention of common viral infections that could be a trigger of CAP (31). The findings of this study are the same as those of Yamamoto et al. showing that the COVID-19 outbreak prevention measures have indirectly contributed to the decrease in the number of pneumonia cases. Influenza and other respiratory viral infections are the most common type of acute respiratory infection. Viral infections predispose the patients to secondary bacterial infections, especially post-influenza secondary bacterial infection. A study by Klein et al. showed the proportion of bacterial pneumonia in patients with influenza that was found to range between 11 and 35% (32). A decline in the incidence of respiratory viral diseases had been reported following the COVID-19 outbreak in the literature. The COVID-19 infection control measures may prevent general viral infections and subsequently bacterial pneumonia.

There are the two COVID-19 screening and treatment center in Chiayi County, Taiwan. Our hospital is one of the two COVID-19 screening and treatment center. We setup the 40 COVID-19 isolation general beds and the 10 COVID-19 intensive care bed during the COVID-19 pandemic. Our hospital provides outpatient and inpatient medical services for general patients and COVID-19 inpatient medical services for the patients with COVID-19. We infer the decrease in the number of pneumonia patients by a reflection of changes in the disease incidence.

### Limitations

The major limitation of this study is that it is a retrospective 2-year observational study at a single hospital and, therefore, can provide only minimal clinical experience. The results may, therefore, not be generalizable owing to different populations of the patient in other hospitals and countries.

## Conclusion

The number of adult patients with CAP in 2020 was lower than that in the same period in 2019 with the number of adult patients with CAP markedly decreasing (−39.7%) in October to December period of 2020. The number of adult patients with HAP in 2020 was lower than that in the same period in 2019 with the number of adult patients with HAP markedly decreasing (−52.4%) in October to December period of 2020. Therefore, interventions applied to control the COVID-19 pandemic were effective not only in influencing substantial changes in the seasonal influenza activity, but also in decreasing pneumonia cases.

## Data Availability Statement

The raw data supporting the conclusions of this article will be made available by the authors, without undue reservation.

## Ethics Statement

The study conformed to the Declaration of Helsinki 1975, revised Hong Kong 1989. The study was approved by the Buddhist Dalin Tzu Chi General Hospital Research Ethics Committee (Approved IRB No. B11002015).

## Author Contributions

The named author meets the International Committee of Medical Journal Editors (ICMJE) criteria for authorship for this article, takes responsibility for the integrity of the work as a whole, and has given their approval for this version to be published.

## Conflict of Interest

The author declares that the research was conducted in the absence of any commercial or financial relationships that could be construed as a potential conflict of interest.

## Publisher's Note

All claims expressed in this article are solely those of the authors and do not necessarily represent those of their affiliated organizations, or those of the publisher, the editors and the reviewers. Any product that may be evaluated in this article, or claim that may be made by its manufacturer, is not guaranteed or endorsed by the publisher.
